# Expression of Epithelial Alarmin Receptor on Innate Lymphoid Cells Type 2 in Eosinophilic Chronic Obstructive Pulmonary Disease

**DOI:** 10.3390/arm92050039

**Published:** 2024-10-18

**Authors:** Katarzyna Królak-Nowak, Marta Wierzbińska, Aleksandra Żal, Adam Antczak, Damian Tworek

**Affiliations:** Department of General and Oncological Pulmonology, Medical University of Lodz, Żeromskiego 113 str., 90-549 Lodz, Poland; katarzyna.krolak-nowak@umed.lodz.pl (K.K.-N.); martawierzbinska@yahoo.com (M.W.); aleksandra.zal@umed.lodz.pl (A.Ż.); adam.antczak@umed.lodz.pl (A.A.)

**Keywords:** eosinophils, epithelial alarmins, COPD, airway inflammation

## Abstract

**Highlights:**

**What are the main findings?**

- We found an increased number of airway ILC2s expressing TSLP receptor and intracellular IL-5 in eosinophilic COPD patients compared with non-eosinophilic patients and heathy smokers.
- In COPD patients’ sputum, ILC2s correlated positively with sputum eosinophilia.

**What is the implication of the main finding?**

- Our report suggests the involvement of ILC2s in airway eosinophilic inflammatory responses in COPD.

**Abstract:**

Studies have shown that eosinophilic COPD (eCOPD) is a distinct phenotype of the disease. It is well established that innate lymphoid cells are involved in the development of eosinophilic inflammation. Interleukin(IL)-25, thymic stromal lymphopoietin (TSLP) and IL-33 are a group of cytokines produced by epithelium in response to danger signals, e.g., cigarette smoke, and potent activators of ILC2s. In the present study, we examined circulating and sputum ILC2 numbers and expression of intracellular IL-5 as well as receptors for TSLP, IL-33 and IL-25 by ILC2s in non-atopic COPD patients with and without (neCOPD) airway eosinophilic inflammation and healthy smokers. In addition, we examined the association between ILC2s and clinical indicators of COPD burden (i.e., symptom intensity and risk of exacerbations). ILC2s were enumerated in peripheral blood and induced sputum by means of flow cytometry. We noted significantly greater numbers of airway IL-5^+^ILC2s and TSLPR^+^ILC2s in eCOPD compared with neCOPD (*p* < 0.05 and *p* < 0.01, respectively) and HSs (*p* < 0.001 for both). In addition, we showed that IL-5^+^ILC2s, IL-17RB^+^ILC2s and ST2^+^ILC2s are significantly increased in the sputum of eCOPD patients compared with HSs. In all COPD patients, sputum ILC2s positively correlated with sputum eosinophil percentage (r = 0.48, *p* = 0.002). We did not find any significant correlations between sputum ILC2s and dyspnea intensity as measured by the modified Medical Research Council scale (mMRC) and symptom intensity measured by the COPD Assessment Test (CAT). These results suggest the involvement of epithelial alarmin-activated ILC2s in the pathobiology of eosinophilic COPD.

## 1. Introduction

A growing body of evidence suggests that eosinophilic COPD is a distinct endotype of the disease. Saetta et al. showed that patients with chronic bronchitis with exacerbation had significantly more eosinophils in their sputum and bronchial biopsies than subjects examined with stable disease [1]. Moreover, earlier studies revealed increased airway eosinophil numbers and eosinophil cationic protein levels (ECP) in a subset of patients with stable, non-atopic COPD [2]. Recently, Kolsum et al. demonstrated that COPD patients with blood eosinophilia had higher eosinophil numbers in their lower airways, which were accompanied by higher airway interleukin(IL)-5, haptoglobin, CCL20 and CCL24 concentrations and more pronounced airway remodeling [3].

It is tempting to speculate that airway epithelial cells might be involved in the development of eosinophilic inflammation in COPD. Activated airway epithelium releases, among other things, three epithelial alarmins, i.e., IL-25, IL-33 and TSLP [4]. A body of evidence supports the notion that IL-25, IL-33 and TSLP are crucial in the pathobiology of allergic asthma and the development and maintenance of eosinophilic airway inflammation. Studies have suggested that IL-33 and TSLP may also be implicated in the pathogenesis of COPD. IL-33 is increased in blood and lung tissues of COPD patients [5,6]. TSLP is overexpressed in COPD airway smooth muscles and produced upon cigarette smoke exposure [7].

Type 2 innate lymphoid cells (ILC2s) constitute cell populations that express receptors for IL-25 (IL-17RB), IL-33 (ST2) and TSLP (TSLPR) [8]. Epithelial alarmins potently activate ILC2s and promote their survival [8,9]. Upon activation, ILC2s produce abundant amounts of IL-5 and are involved in the development of persistent airway eosinophilia in asthma despite treatment with systemic steroids [10]. Some studies showed that although frequencies of circulating and lung ILC2s are decreased in COPD, this cell population may still play a significant role in the activation of eosinophils in COPD [11,12]. However, ILC2s in eosinophilic COPD have not been examined so far.

The aim of the present study was to (1) assess numbers of ILC2s and (2) determine epithelial alarmin receptor expression on ILC2s in blood and induced sputum in non-atopic COPD subjects with and without sputum eosinophilia. In addition, we examined the association between ILC2s and clinical indicators of COPD burden (i.e., symptom intensity and risk of exacerbations).

## 2. Materials and Methods

### 2.1. Study Population

Patients with COPD who were diagnosed according to Global Initiative for Lung Obstructive Disease (GOLD) criteria were eligible for this study [13]. All subjects had to be non-atopic based on skin prick test (SPT) results. Patients were classified as having eosinophilic COPD (eCOPD) or non-eosinophilic COPD (neCOPD) based on the percentage of eosinophils in induced sputum. Patients were inhaled steroid-naïve and treated with either mono- or dual bronchodilation. Sputum eosinophilia was defined as a sputum eosinophil percentage >3%. The control group comprised healthy current smokers (HSs) with normal spirometry. None of the study participants had airway infections or COPD exacerbation within 4 weeks before the study visit. Subjects were not eligible for the study if they had a history of atopy, allergic asthma or allergic rhinitis or used inhaled, nasal or oral steroids within 4 weeks before the study visit.

### 2.2. The COPD Assessment Test (CAT)

The CAT is a simple, validated health status instrument for patients with COPD. The self-administered questionnaire consists of eight items assessing various manifestations of COPD and the global impact of the disease on health status. It is a simple, quantified measure of health-related quality of life. CAT scores range from 0 to 40. A decrease in CAT score represents an improvement in health status, whereas an increase in CAT score represents a worsening in health status [14].

### 2.3. The Modified Medical Research Council (mMRC) Dyspnea Scale

mMRC is a five-level rating scale based on the patient’s perception of dyspnea in daily activities. It consists of five statements that describe the entire range of dyspnea from none (Grade 0) to almost complete incapacity (Grade 4) [15].

### 2.4. Symptom Intensity and Risk of Exacerbations

Patients were categorized into four groups according to symptom intensity and risk of exacerbation in line with GOLD definitions [13]. The low symptom intensity groups (patient groups A and C) comprised patients with CAT scores <10 and mMRC of 0–1 points. Highly symptomatic patients had CAT scores ≥10 or mMRC ≥2 points (patient groups B and D). Low risk of exacerbation was defined as a history of ≤1 exacerbation in the last 12 months not leading to hospitalization (patient groups A and B). High exacerbation risk was defined as ≥2, moderate (need for use of oral steroids and/or antibiotics), or ≥1, severe (leading to hospital admission), exacerbation history within the last 12 months (patient groups C and D).

### 2.5. Skin Prick Testing

All patients underwent skin prick tests performed with common aeroallergens: Dermatophagoides pteronyssinus, Dermatophagoides farinae, grasses, birch, hazel, alder, mugwort, cat, dog, Alternaria tenuis and Cladosporium herbarum (Allergopharma, Reinbek, Germany). Histamine 1.7 mg/mL (Allergopharma) and standard glycerol saline solution (Allergopharma) were used as positive and negative controls, respectively. A wheal diameter ≥3 mm was considered a positive result.

### 2.6. Blood Sample Processing

Peripheral venous blood samples were withdrawn into lithium heparin tubes (BD Dickenson, Franklin Lakes, NJ, USA). This blood was diluted with McCoys 5A (Invitrogen, Waltham, MA, USA) and then layered on Lymphoprep (d = 1.077 mg/mL; Axis-Shield, Dundee, UK) and centrifuged at 2200 rpm for 20 min at room temperature. Peripheral blood mononuclear cells (PBMCs) were removed and washed with McCoy 5A (centrifugation at 1500 rpm for 10 min at 4 °C). Two million PBMCs per tube were used for staining for flow cytometry.

### 2.7. Sputum Induction and Processing

Sputum samples were induced using hypertonic saline. Selected mucous plugs were processed using a two-step method with a Dulbecco’s phosphate-buffered saline (D-PBS) wash step followed by a dithiothreitol (DTT) step and cytospins [16]. Cytospins were prepared from sputum cells and stained with Diff-Quik for differential cell counts. The remaining cells were subjected to flow cytometry staining.

### 2.8. Flow Cytometry

**Figure 1:**
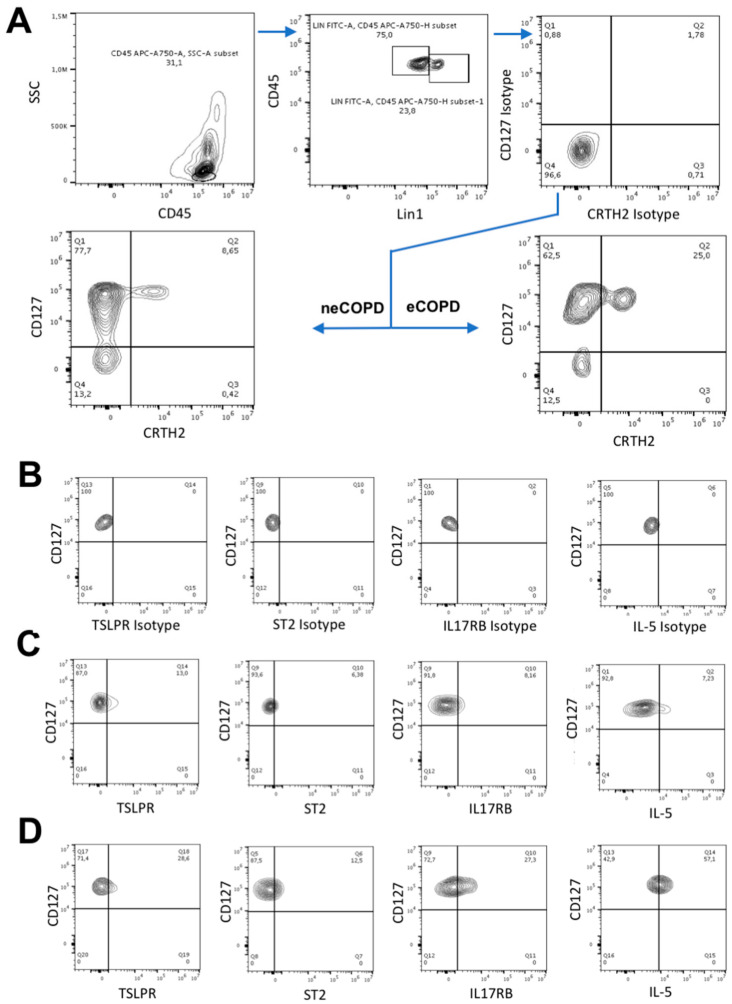
Innate lymphoid cell type 2 (ILC2) gating strategy. Freshly isolated cells were immunostained with anti-CD45, Lineage cocktail 1 (Lin1) and either anti-CD127 and anti-CRTH2 or respective isotype antibodies to acquire the ILC2 population (**A**). Evaluation of thymic stromal lymphopoietin receptor (TSLPR), interleukin(IL)-33 receptor (ST2), IL-25 receptor (IL-17RB) and intracellular IL-5 expression was performed based on unspecific isotype (**B**) and specific antibody staining. (**C**)—Representative sputum sample of non-eosinophilic COPD patient and (**D**)—representative sputum sample of eosinophilic COPD patient.

PBMC and sputum cells for flow cytometry experiments were immunostained with isotype or specific antibodies to the extracellular CD45 (BD Biosciences, Franklin Lakes, NJ, USA), Lineage cocktail 1 (anti-CD3, -CD14, -CD16, -CD19, -CD20, and -CD56) (BD Biosciences), FceR1α (Invitrogen), CRTH2 (BD Biosciences), CD127 (Invitrogen), TSLPR (BD Biosciences), IL17RB (R&D) and ST2 (R&D). To measure intracellular IL-5 expression, cells were washed, fixed and permeabilized, then stained with isotype control or antibody to IL-5 (R&D). Cells were washed and acquired with a CytoFLEX flow cytometer (Beckman Coulter, Brea, CA, USA). Analyses were performed using Flow-Jo software (Tree Star, Ashland, OR, USA). ILC2s were defined as a CD45^+^Lin^−^CRTH2^+^CD127^+^ population according to the previously described gating strategy (Figure 1) [17]. The isotype control for the markers of interest was set to 2%, which was compared to the specific markers to detect the percentage of cells expressing the marker.

### 2.9. Statistical Analysis

The results were analyzed using GraphPad Prism 6 (GraphPad Software, San Diego, CA, USA). For clarity, all results are presented as means ± SEMs. The normality of data distribution was tested with the Shapiro–Wilk test. Comparisons between neCOPD patients, eCOPD patients and healthy smokers were made with one-way ANOVA and the post hoc Tukey’s test, or with the Kruskal–Wallis test and the post hoc Dunn’s test, when appropriate.

Differences between COPD subjects with and without sputum eosinophilia were analyzed using the Student’s *t*-test or Mann–Whitney U test, when appropriate. Correlation analysis was conducted using either Pearson’s or Spearman’s correlation coefficient according to the distribution of variables. Significance was accepted at *p* < 0.05.

## 3. Results

### 3.1. Study Population

Thirty-seven patients with COPD and ten non-atopic HSs were included. Twenty patients had neCOPD and seventeen had eCOPD based on sputum eosinophil counts.

Subject characteristics are presented in Table 1.

### 3.2. Circulating ILC2s

There was no difference in circulating ILC2s between the HS, eCOPD and neCOPD groups (see Supplementary Figure S1A) or in ILC2s expressing intracellular IL-5, ST-2 and IL-17RB. However, we found increased numbers of TSLPR^+^ILC2s in eCOPD compared with neCOPD (10.10 ± 2.19 cells/1 mln of PBMC vs. 2.29 ± 1.28 cells/1 mln of PBMC, respectively; *p* < 0.05) (see Supplementary Figure S1C). For details, see the Supplementary Materials online.

### 3.3. Sputum ILC2s

**Figure 2:**
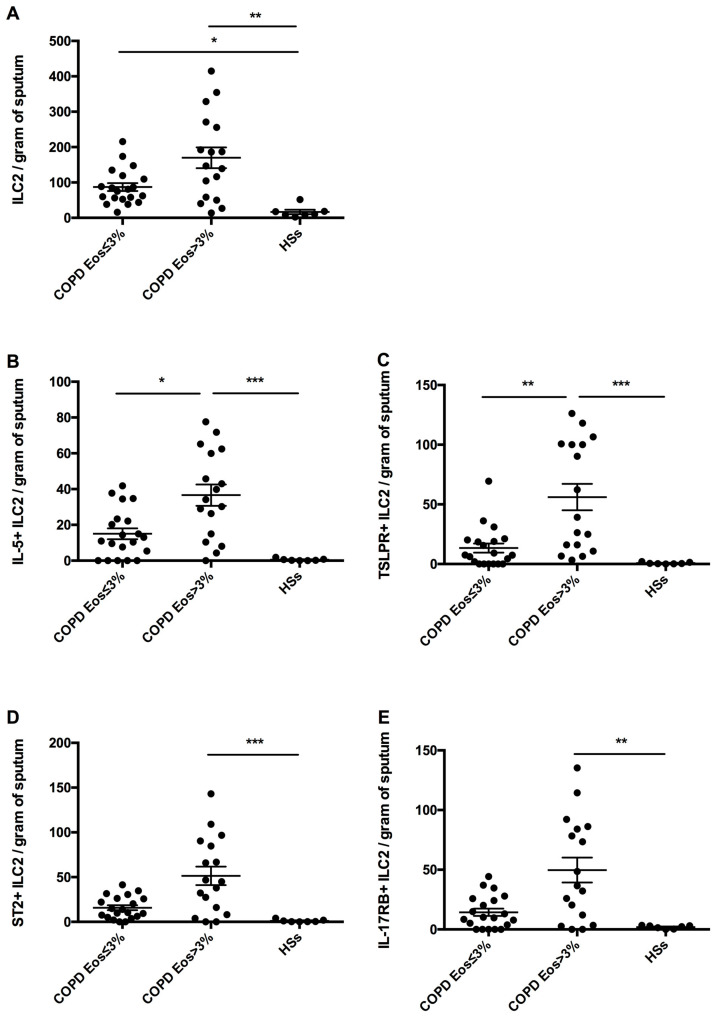
Sputum innate lymphoid cell type 2 (ILC2) numbers (subfigure (**A**)) and sputum ILC2s expressing intracellular interleukin(IL)-5 (subfigure (**B**)), thymic stromal lymphopoietin receptor (TSLPR) (subfigure (**C**)), IL-33 receptor (ST2) (subfigure (**D**)) and IL-25 receptor (IL-17RB) (subfigure (**E**)) in neCOPD patients (Eos < 3%), eCOPD patients (Eos > 3%) and healthy smokers (HSs). * *p* < 0.05; ** *p* <0.01; *** *p* < 0.001.

Sputum ILC2 numbers were significantly lower in HSs (16.75 ± 6.24 cells/g of sputum) compared with the neCOPD (87.07 ± 11.21 cells/g of sputum; *p* < 0.05) and eCOPD (169.80 ± 29.42 cells/g of sputum; *p* < 0.01) groups (Figure 2A).

IL-5+ILC2s were significantly lower in HSs (0.54 ± 0.24 cells/g of sputum) compared with eCOPD patients (36.64 ± 5.95 cells/g of sputum; *p* < 0.001) but not neCOPD patients (15.03 ± 3.03 cells/g of sputum) (Figure 2B).

There were significantly fewer sputum TSLPR^+^ILC2s in HSs (0.75 ± 0.28 cells/g of sputum) compared with eCOPD patients (56.11 ± 11.09 cells/g of sputum; *p* < 0.001) but not neCOPD patients (13.40 ± 3.82 cells/g of sputum; *p* > 0.05) (Figure 2C). Sputum ST2+ILC2 numbers were also significantly higher in eCOPD patients (51.42 ± 10.27 cells/g of sputum; *p* < 0.001) but not neCOPD patients (15.80 ± 2.79 cells/g of sputum; *p* > 0.05) compared with HSs (1.15 ± 0.55 cells/g of sputum) (Figure 2D). A similar pattern was noted for IL-17RB^+^ILC2s (eCOPD: 49.74 ± 10.47 cells/g of sputum vs. HSs: 1.93 ± 0.52 cells/g of sputum; *p* < 0.01; and neCOPD: 14.37 ± 3.08 cells/g of sputum vs. HSs; *p* > 0.05).

In addition, sputum IL-5^+^ILC2 and TSLPR^+^ILC2 numbers were significantly higher in eCOPD compared with neCOPD patients (*p* < 0.05 and *p* < 0.01, respectively). There was no significant difference in sputum ILC2, ST2^+^ILC2 and IL-17RB^+^ILC2 numbers between neCOPD and eCOPD patients (*p* > 0.05 for all).

### 3.4. Circulating ILC2 Numbers According to Risk of Exacerbations and Symptom Intensity

There was no significant difference in circulating ILC2s between low and high risk of exacerbation (39.99 ± 4.29 cells/1 mln PBMC and 53.64 ± 24.97 cells/1 mln PBMC, respectively; *p* = 0.95) (see Supplementary Figure S2A) or between low symptom intensity and highly symptomatic (44.31 ± 7.28 cells/1 mln PBMC and 41.58 ± 5.64 cells/1 mln PBMC, respectively; *p* = 0.76) (see Supplementary Figure S2B) for all COPD subjects.

There was no significant difference in circulating ILC2s between symptomatic and asymptomatic neCOPD patients (36.47 ± 5.96 cells/1 mln of PBMC vs. 50.38 ± 8.87 cells/1 mln PBMC) (see Supplementary Figure S2E). However, a higher number of circulating ILC2s in highly symptomatic eCOPD patients compared to the eCOPD low symptom intensity group (207.1 ± 34.63 cells/1 mln PBMC vs. 80.24 ± 31.83 cells/1 mln PBMC, respectively; *p* = 0.036) was noted (see Supplementary Figure S2F).

There was no significant difference in the number of circulating ILC2s expressing TSLPR, ST2, IL-17RB and intracellular IL-5 between the low and high symptom intensity groups in either neCOPD or eCOPD patients (see Supplementary Figures S3–S6).

Patient numbers were too low to perform separate analyses for the neCOPD and eCOPD groups depending on the risk of exacerbations (see Supplementary Figure S2C,D).

### 3.5. Sputum ILC2 Numbers According to Risk of Exacerbations and Symptom Intensity

**Figure 3:**
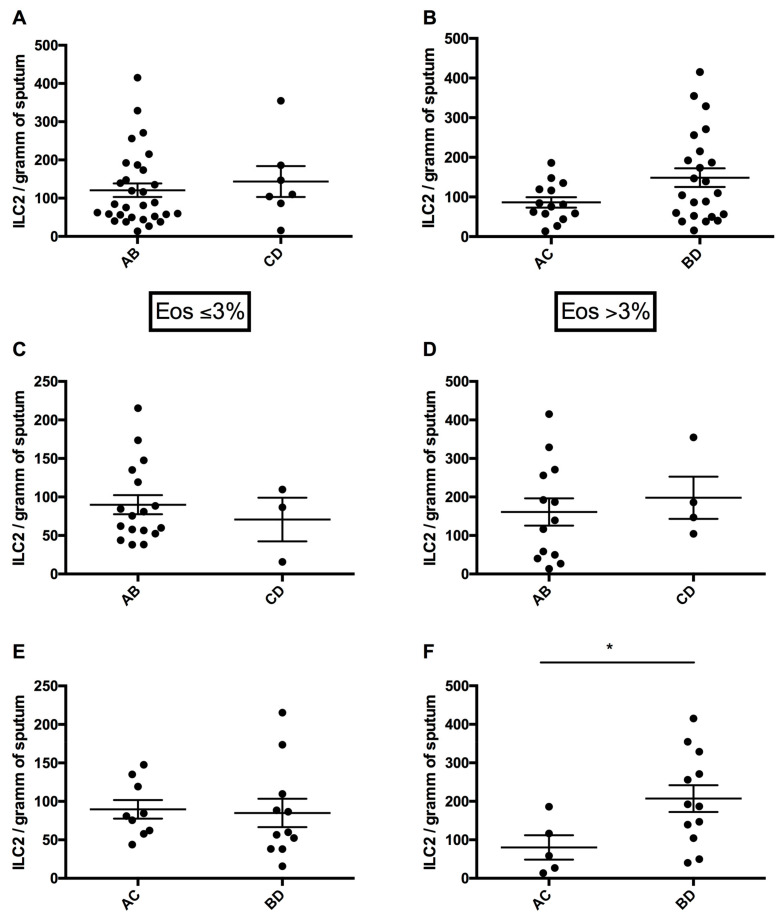
Sputum innate lymphoid cells type 2 (ILC2s) depending on symptom level and exacerbation risk in all COPD (subfigures (**A**,**B**)), neCOPD (Eos ≤ 3%) (subfigures (**C**,**E**)) and eCOPD (Eos > 3%) (subfigures (**D**,**F**)) patients. AB—patients at low risk of exacerbations; CD—patients at high risk of exacerbations; AC—patients with low symptom intensity; BD—patients with high symptom intensity (as defined by GOLD). * *p* < 0.05.

Although sputum ILC2 numbers were increased in highly symptomatic COPD patients (148.70 ± 23.57 cells/g of sputum) compared with the low symptom intensity group (86.28 ± 13.05 cells/g of sputum), this difference did not reach statistical significance (*p* = 0.16) (Figure 3B). Moreover, there was no significant difference between COPD subjects with low (120.80 ± 17.76 cells/g of sputum) and high (143.40 ± 40.42 cells/g of sputum; *p* = 0.48) risk of exacerbation (Figure 3A).

In neCOPD patients, we did not find differences in ILC2 numbers between low- and highly symptomatic subjects. However, in eCOPD, we noted significantly greater numbers of sputum ILC2s in highly symptomatic patients (207.10 ± 34.63 cells/g of sputum) compared with the low symptom intensity group (80.24 ± 31.83 cells/g of sputum; *p* = 0.04) (Figure 3F).

**Figure 4:**
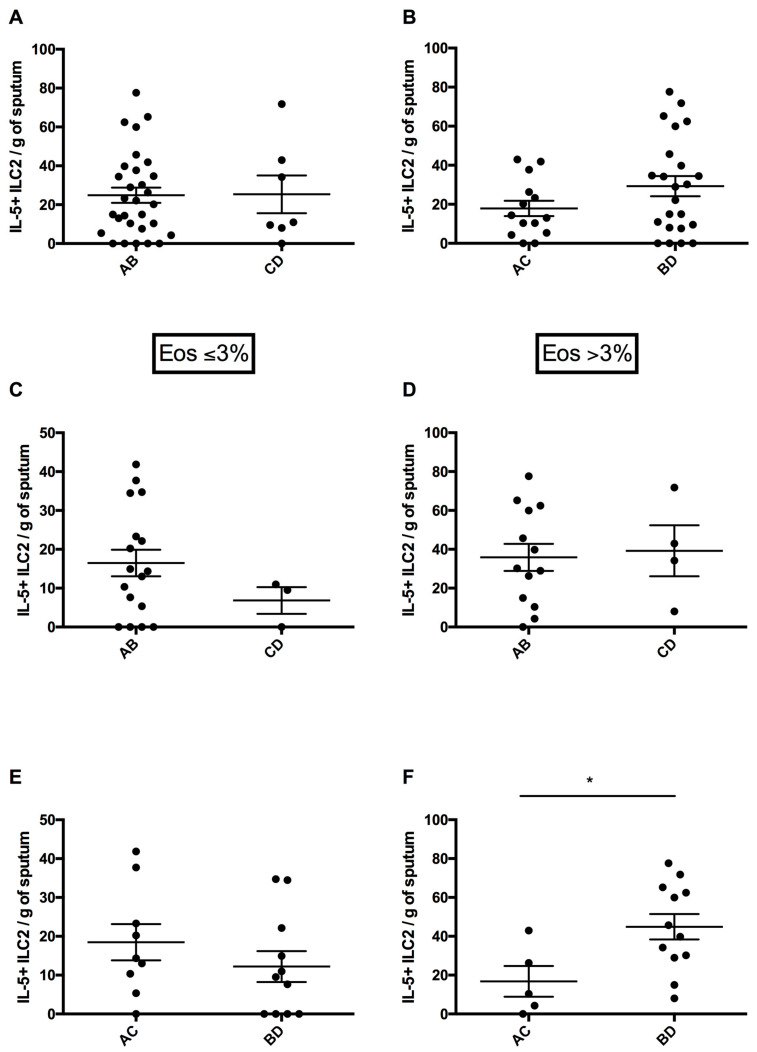
Sputum innate lymphoid cells type 2 (ILC2s) expressing intracellular IL-5 depending on symptom level and exacerbation risk in all COPD (subfigures (**A**,**B**)), neCOPD (Eos < 3%) (subfigures (**C**,**E**)) and eCOPD (Eos > 3%) (subfigures (**D**,**F**)) patients. AB—patients at low risk of exacerbations; CD—patients at high risk of exacerbations; AC—patients with low symptom intensity; BD—patients with high symptom intensity (as defined by GOLD). * *p* < 0.05.

In parallel with circulating ILC2s, there was no significant difference in the number of sputum ILC2s expressing intracellular TSLPR, ST2 and IL-17RB between the low and high symptom intensity groups in either neCOPD or eCOPD patients. Nonetheless, we noted significantly more sputum IL5^+^ILC2s in highly symptomatic eCOPD patients (44.91 ± 6.51 cells/g of sputum) compared with the low symptom group (16.80 ± 7.91 cells/g of sputum; *p* = 0.02) (Figure 4F).

Patient numbers were too low to perform separate analyses for the neCOPD and eCOPD groups depending on the risk of exacerbations (Figure 4C,D).

### 3.6. Correlation Between Sputum ILC2s and Sputum Eosinophils and mMRC and CAT

**Figure 5:**
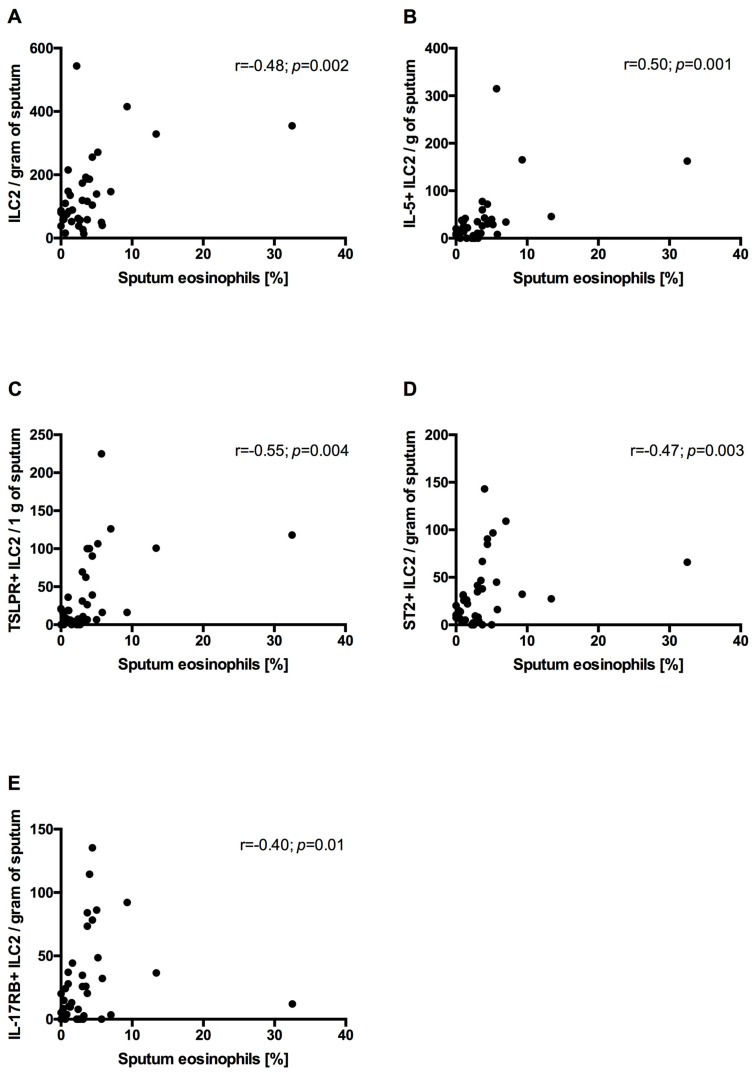
Correlation between sputum eosinophils and sputum innate lymphoid cells type 2 (ILC2s) (**A**) and ILC2s expressing intracellular IL-5 (**B**), TSLPR (**C**), ST2 (**D**) and IL-17RB (**E**) in all COPD subjects.

In all COPD patients, the sputum eosinophil percentage positively correlated with sputum ILC2s (r = 0.48; *p* = 0.002), IL-5^+^ILC2s (r = 0.50; *p* = 0.001), TSLPR+ILC2s (r = 0.55; *p* = 0.004), ST2+ILC2s (r = 0.47; *p* = 0.003) and IL-17RB+ILC2s (r = 0.40; *p* = 0.01) (Figure 5).

**Figure 6:**
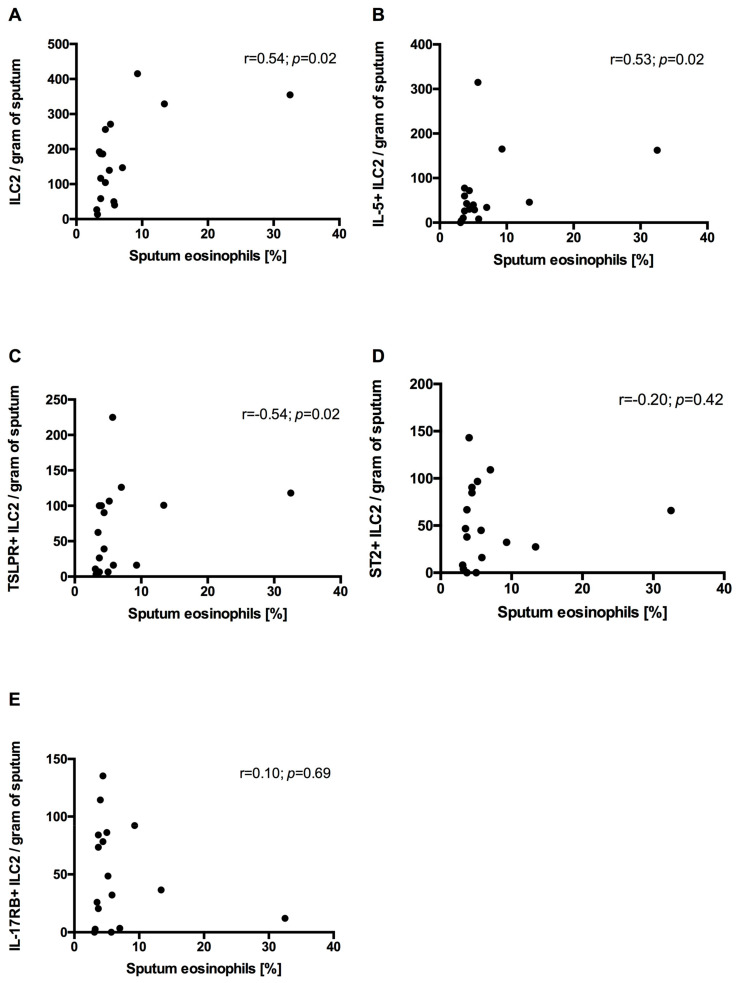
Correlation between sputum eosinophils and sputum innate lymphoid cells type 2 (ILC2s) (**A**) and ILC2s expressing intracellular IL-5 (**B**), TSLPR (**C**), ST2 (**D**) and IL-17RB (**E**) in eosinophilic COPD subjects.

In neCOPD sputum, eosinophils did not correlate with either sputum ILC2s or ILC2s expressing intracellular IL-5 and epithelial alarmin receptors. Nevertheless, in the eCOPD group the sputum eosinophil percentage positively correlated with ILC2s, IL-5+ILC2s and TSLPR+ILC2s but not ILC2s expressing ST2 and IL-17RB (Figure 6).

We did not find any correlations between sputum ILC2s and mMRC and CAT (see Supplementary Table S1).

## 4. Discussion

To our knowledge, this is the first study assessing ILC2s in non-atopic eosinophilic COPD. We demonstrated that ILC2s are increased in the sputum of eCOPD patients, in particular in those classified as symptomatic according to GOLD (therapeutic groups B and D). In addition, ILC2s correlated with a degree of airway eosinophilic inflammation.

The eosinophilic endotype may be persistently present in more than one-third of COPD patients when a blood eosinophil percentage of ≥2% is applied to define eCOPD [18]. The mechanism leading to development of eosinophilic infiltration of the airways in some COPD subjects is not well understood. Moreover, knowledge on the differences in systemic and airway inflammation patterns between eosinophilic and non-eosinophilic COPD is scanty. In a recent study, Kolsum at al. assessed the nature of airway inflammation in patients with COPD based on blood eosinophil numbers [3]. The authors classified study participants based on blood eosinophil counts as “eosinophil low” or “eosinophil high” (<150 cells/μL or >250 cells/μL, respectively). Higher sputum, bronchoalveolar lavage (BAL) and bronchial submucosa eosinophil numbers were observed in COPD patients with blood eosinophils >250 cells/μL. This was accompanied by higher sputum IL-5 and haptoglobin levels as well as CCL20 and CCL24 concentrations in BAL. “Eosinophil high” subjects presented increased reticular basement thickness and tenascin thickness as well as increased BAL metalloproteinase-7 and -9 concentrations, i.e., indicators of exaggerated airway remodeling.

Data on ILC2s in COPD are limited and conflicting. Importantly, published reports do not relate ILC2 levels to allergic/atopic status nor stratified COPD patients based on eosinophilic endotype. It appears that the circulating ILC2 percentage is decreased in COPD, in favor of the ILC1 population [10]. On the contrary, Jiang et al. found an increased proportion of circulating ILC2s in COPD patients compared to HSs [19]. Others showed similar frequencies of lung ILC2s in COPD GOLD stage I-II patients and HSs [20]. Another study showed in a small number of patients that lung ILC2 frequency is lower in COPD GOLD stage I-II patients relative to healthy subjects, and a diminished number of ILC2s results from IL-12 overproduction in COPD [12]. However, the same study demonstrated the eosinophil–ILC2 interplay resulting in the activation of not only the eosinophils but also ILC2s. Therefore, it is possible that ILC2s might have a role in the development of the eosinophilic COPD endotype. Our study showed increased absolute numbers of sputum ILC2s in neCOPD and eCOPD. Specifically, ILC2 numbers expressing IL-5 and alarmin receptors were increased in the sputum of eCOPD subjects compared with healthy smokers. Moreover, IL-5+ILC2s and TSLPR+ILC2s correlated positively with sputum eosinophil percentage. It is plausible that TSLP-stimulated ILC2s are involved in fostering eosinophilic inflammation by producing IL-5, a main cytokine implicated in eosinophil activation.

The airway lumen is exposed to the environment, and epithelial cells respond to many factors, e.g., allergens but also infections and cigarette smoke (CS). As a result, activated airway epithelium releases IL-25, IL-33 and TSLP. Several studies have shown that these cytokines play a key role in the development of eosinophilic inflammation in allergic diseases [21,22,23]. In addition, data show that epithelial alarmins are implicated in COPD pathobiology. For instance, Nakamura et al. reported that repeated intranasal exposure to cigarette smoke extract induced TSLP mRNA and protein expression in the mouse lung [24]. Other experiments demonstrated that human airway smooth muscles (ASMs) are an important source of TSLP [25]. Moreover, expression of both TSLP and TSLPR as well as TSLP release by ASMs increase following exposure to cigarette smoke extract [7].

In one of our previous studies, we showed that IL-33 may promote airway eosinophilia in COPD patients through stimulation of in situ differentiation of hematopoietic precursor cells into eosinophils [6]. Furthermore, there is evidence that IL-33 promotes proliferation of ILC2s and secretion of IL-4, IL-5, IL-6 and IL-13 [19]. In the present study, we showed that the numbers of ILC2s expressing alarmin receptors and intracellular IL-5 are significantly higher in eCOPD patients compared with healthy smokers. In addition, TSLPR+ILC2 and IL5+ILC2 numbers are increased in eCOPD compared with neCOPD. This finding suggests that activation of ILC2s by TSLP may be another link in the epithelial alarmin–airway eosinophilia axis.

We also demonstrated that ILC2s and IL-5+ILC2s are increased in highly symptomatic eCOPD patients, indicating that ILC2s may be involved in sustaining airway inflammation and thus respiratory symptoms in these subjects. There is evidence that treatment of allergic asthmatics with inhaled steroids was able to reverse the high levels of IL-5, IL-13 and IL-9 produced by ILC2s via STAT3, STAT5, STAT6, JAK3 and MEK signaling pathways, which was accompanied by better asthma control [26]. In parallel, our findings suggest that symptomatic eCOPD patients may benefit from treatment with inhaled steroids as it may improve respiratory symptoms by reducing ILC2 numbers, thus diminishing airway inflammation. However, it was also demonstrated that TSLP may induce steroid resistance in ILC2s [27]. Therefore, increased expression of TSLPR on ILC2s may lead to steroid resistance in airway ILC2s. A study examining responses to inhaled steroids depending on TSLPR expression in ILC2s is warranted to clarify this issue.

Our study has some limitations. Firstly, the control group of smokers without COPD and the subgroup of patients at high risk of exacerbation (GOLD groups C and D) are relatively small, which might have prevented us from finding significant differences between study groups. Moreover, we did not include a group of healthy non-smokers into our study since our main goal was to study airway inflammation and it was previously shown that low cell recovery from induced sputum precludes ILC2 analyses in healthy controls [28]. Last but not least, we did not measure blood eosinophils and did not analyze ILC2s and epithelial alarmin receptor expression in respect to blood eosinophilia. It was previously shown in the SPIROMICS study that blood eosinophils do not reliably predict airway eosinophils in induced sputum and are poor predictors of clinical outcomes in COPD patients [29]. Therefore, our focus was on sputum eosinophilia and airway inflammation.

## 5. Conclusions

In conclusion, our report suggests involvement of ILC2s in airway eosinophilic inflammatory response in a subset of COPD patients. We speculate that airway ILC2s may be activated by epithelial alarmins, in particular TSLP, i.e., cytokines associated with Th2 responses but produced by epithelial cells upon activation not only by allergens but also other danger signals, for instance, cigarette smoke. Further studies in symptomatic COPD patients with eosinophilic airway inflammation are warranted to assess treatment responses to inhaled corticosteroids, which are, for the time being, recommended mainly in patients with a high risk of exacerbations.

## Figures and Tables

**Table 1:** Study participant demographics. NA—not applicable. PB—post bronchodilator. * eCOPD vs. neCOPD and eCOPD vs. HSs, for both comparisons *p* < 0.001; ** HSs vs. neCOPD and eCOPD, for both comparisons *p* < 0.01; *** HSs vs. neCOPD, *p* < 0.05; **** HSs vs. neCOPD and eCOPD, for both comparisons *p* < 0.001.

	neCOPD	eCOPD	HSs
Sex (number of M/F)	11/9	10/7	5/5
Age (years)	62.15 ± 1.75	57.47 ± 1.91	58.50 ± 2.44
Number of pack-years	32.60 ± 3.56	29.12 ± 3.03	26.40 ± 4.02
Smoking status (smoker/ex-smoker)	16/4	13/4	10/0
Sputum eosinophil count (%)	1.3 ± 0.22	6.91 ± 1.71 *	1.02 ± 0.37
Sputum macrophage count (%)	30.30 ± 3.17	30.45 ± 4.11	53.14 ± 4.61 **
Sputum neutrophil count (%)	57.44 ± 4.35	53.65 ± 4.79	38.70 ± 4.31 ***
Sputum lymphocyte count (%)	3.28 ± 0.30	2.85 ± 0.56	2.37 ± 0.37
Number of atopic subjects	0	0	0
PB FEV_1_ (% of predicted)	71.37 ± 4.53	74.20 ± 8.92	92.36 ± 6.41 ****
PB FEV_1_%FVC	57.29 ± 7.62	61.72 ± 6.85	75.17 ± 2.48 ****
mMRC (points, median (min–max))	1 (0–3)	1 (0–3)	NA
CAT (score)	12.40 ± 1.58	15.88 ± 1.93	NA

## Data Availability

The original contributions presented in this study are included in the article/Supplementary Materials; further inquiries can be directed to the corresponding author/s.

## Supplementary Materials

The following supporting information can be downloaded at https://www.mdpi.com/article/10.3390/arm92050039/s1: Supplementary Materials; Figure S1: Circulating innate lymphoid cell type 2 (ILC2) numbers and circulating ILC2s expressing intracellular interleukin(IL)-5, thymic stromal lymphopoietin receptor (TSLPR), IL-33 receptor (ST2) and IL-25 receptor (IL-17RB); Figure S2: Circulating innate lymphoid cells type 2 (ILC2s) depending on symptom level and exacerbation risk in all COPD, neCOPD (Eos < 3%) and eCOPD (Eos > 3%) patients.; Figure S3: Circulating innate lymphoid cells type 2 (ILC2s) expressing thymic stromal lymphopoietin receptor (TSLPR) depending on symptom level and exacerbation risk in all COPD, neCOPD (Eos < 3%) and eCOPD (Eos > 3%) patients; Figure S4: Circulating innate lymphoid cells type 2 (ILC2s) expressing interleukin(IL)-33 receptor (ST2) depending on symptom level and exacerbation risk in all COPD, neCOPD (Eos < 3%) and eCOPD (Eos > 3%) patients; Figure S5: Circulating innate lymphoid cells type 2 (ILC2s) expressing interleukin(IL)-25 receptor (IL-17RB) depending on symptom level and exacerbation risk in all COPD, neCOPD (Eos < 3%) and eCOPD (Eos > 3%) patients; Figure S6: Circulating innate lymphoid cells type 2 (ILC2s) expressing intracellular interleukin(IL)-5 receptor depending on symptom level and exacerbation risk in all COPD, neCOPD (Eos < 3%) and eCOPD (Eos > 3%) patients; Table S1: Correlations between innate lymphoid cells type 2 (ILC2s) and COPD Assessment Test (CAT) and modified Medical Research Council (mMRC) dyspnea scale. IL-5—intereleukin-5; TSLPR—thymic stromal lymphopoietin receptor; ST2—IL-33 receptor; IL-17RB—IL-25 receptor.
