# Supplementary material for: Expression of Epithelial Alarmin Receptor on Innate Lymphoid Cells Type 2 in Eosinophilic Chronic Obstructive Pulmonary Disease
Source: Adv Respir Med. 2024 Oct 18;92(5):429–43. doi: 10.3390/arm92050039 (PMC11505438; doi:10.3390/arm92050039)
Supplement: Supplementary file 1 [file arm-92-00039-s001.zip › arm-3217504-supplementary.pdf]

advances in respiratory medicine

MDPI

Expression of Epithelial Alarmin Receptor on Innate Lymphoid Cells Type 2 in Eosinophilic CHRONIC Obstructive Pulmonary Disease

Circulating ILC2s

There was no significant difference between the neCOPD, eCOPD and HS groups (37,89±4,58 cells/1mln PBMC vs. 37,32±4,45 cells/1mln PBMC vs. 26,44±3,39 cells/1mln PBMC, respectively; p>0.05) (Supplementary Figure 1A). In addition, we did not note significant differences between neCOPD and eCOPD in circulating ILC2s expressing intracellular IL-5 (12,79±2,96 cells/1mln PBMC vs. 19,09±3,75 cells/1 mln PBMC, respectively; p=0,16), ST2 (2,89±0,63 cells/1mln PBMC vs. 3,66±0,52 cells/1 mln PBMC, respectively; p=0,65) and IL17-RB (1,45±0,30 cells/1mln PBMC vs. 1,87±0,53 cells/1 mln PBMC, respectively; p=0,93) (Supplementary Figure 1B, 1D, 1E, 1F). Circulating ILC2s expressing TSLPR were increased in eCOPD compared with neCOPD (10,10±2,19 cells/1 mln PBMC vs. 3,17±0,71 cells/1mln PBMC, respectively; p=0,93) (Supplementary Figure 1E).

Supplementary Tables:

Table S1. Correlations between innate lymphoid cells type 2 (ILC2s) and COPD Assessment Test (CAT) and modified Medical Research Council (mMRC) dyspnea scale. IL-5—intereleukin-5; TSLPR—thymic stromal lymphopoietin receptor; ST2—IL-33 receptor; IL-17RB—IL-25 receptor.

	ILC2	ILC2IL-5+	ILC2TSLPR+	ILC2ST2+	ILC2IL-17RB+
**Blood**					
**All COPD patients**					
CAT	r=0,17; p=0,29	r=0,00; p=0,99	r=0,15; p=0,35	r=0,12; p=0,47	r=0,03; p=0,83
mMRC	r=0,03; p=0,84	r=0,06; p=0,71	r=-0,01; p=0,95	r=0,02; p=0,87	r=0,03; p=0,85
**neCOPD**					
CAT	r=-0,06; p=0,78	r=-0,27; p=0,23	r=-0,24; p=0,30	r=-0,06; p=0,77	r=0,07; p=0,73
mMRC	r=-0,16; p=0,49	r=-0,06; p=0,77	r=0,24; p=0,28	r=0,07; p=0,73	r=0,19; p=0,40
**eCOPD**					
CAT	r=-0,03; p=0,88	r=0,04; p=0,85	r=-0,05; p=0,83	r=0,21; p=0,41	r=0,07; p=0,78
mMRC	r=-0,21; p= 0,04	r=0,15; p=0,55	r=-0,35; p=0,15	r=-0,03; p=0,89	r=-0,26; p=0,29
**Induced Sputum**					
**All COPD patients**					
CAT	r=0,11; p=0,50	r=0,11; p=0,48	r=0,08; p=0,62	r=0,20; p=0,62	r=0,21; p=0,20
mMRC	r=0,04; p=0,80	r=0,11; p=0,50	r=0,12; p=0,45	r=0,05; p=0,72	r=0,21; p=0,19
**neCOPD**					
CAT	r=-0,07; p=0,73	r=-0,05; p=0,82	r=-0,16; p=0,49	r=0,09; p=0,70	r=-0,02; p=0,92
mMRC	r=-0,00; p=0,97	r=0,01; p=0,95	r=0,18; p=0,43	r=0,18; p=0,42	r=-0,04; p=0,86
**eCOPD**					
CAT	r=-0,03; p=0,88	r=0,04; p=0,86	r=-0,05; p=0,84	r=0,11; p=0,65	r=0,23; p=0,36
mMRC	r=-0,16; p=0,52	R=0,09; P=0,71	R=-0,19; P=0,45	R=-0,17; P=0,50	R=0,36; P=0,14

Supplementary Figure Legends

Figure S1. Circulating innate lymphoid cell type 2 (ILC2) numbers and circulating ILC2s expressing intracellular interleukin(IL)-5, thymic stromal lymphopoietin receptor (TSLPR), IL-33 receptor (ST2) and IL-25 receptor (IL-17RB).

Figure S2. Circulating innate lymphoid cells type 2 (ILC2s) depending on symptom level and exacerbation risk in all COPD, neCOPD (Eos<3%) and eCOPD (Eos>3%) patients.

Figure S3. Circulating innate lymphoid cells type 2 (ILC2s) expressing thymic stromal lymphopoietin receptor (TSLPR) depending on symptom level and exacerbation risk in all COPD, neCOPD (Eos<3%) and eCOPD (Eos>3%) patients.

Figure S4. Circulating innate lymphoid cells type 2 (ILC2s) expressing interleukin(IL)-33 receptor (ST2) depending on symptom level and exacerbation risk in all COPD, neCOPD (Eos<3%) and eCOPD (Eos>3%) patients.

Figure S5. Circulating innate lymphoid cells type 2 (ILC2s) expressing interleukin(IL)-25 receptor (IL-17RB) depending on symptom level and exacerbation risk in all COPD, neCOPD (Eos<3%) and eCOPD (Eos>3%) patients.

Figure S6. Circulating innate lymphoid cells type 2 (ILC2s) expressing intracellular interleukin(IL)-5 receptor depending on symptom level and exacerbation risk in all COPD, neCOPD (Eos<3%) and eCOPD (Eos>3%) patients.
