# Supplementary material for: Expression of Epithelial Alarmin Receptor on Innate Lymphoid Cells Type 2 in Eosinophilic Chronic Obstructive Pulmonary Disease
Source: Adv Respir Med. 2024 Oct 18;92(5):429–43. doi: 10.3390/arm92050039 (PMC11505438; doi:10.3390/arm92050039)
Supplement: Supplementary file 1 [file arm-92-00039-s001.zip › Supplementary Figure 4.pdf]

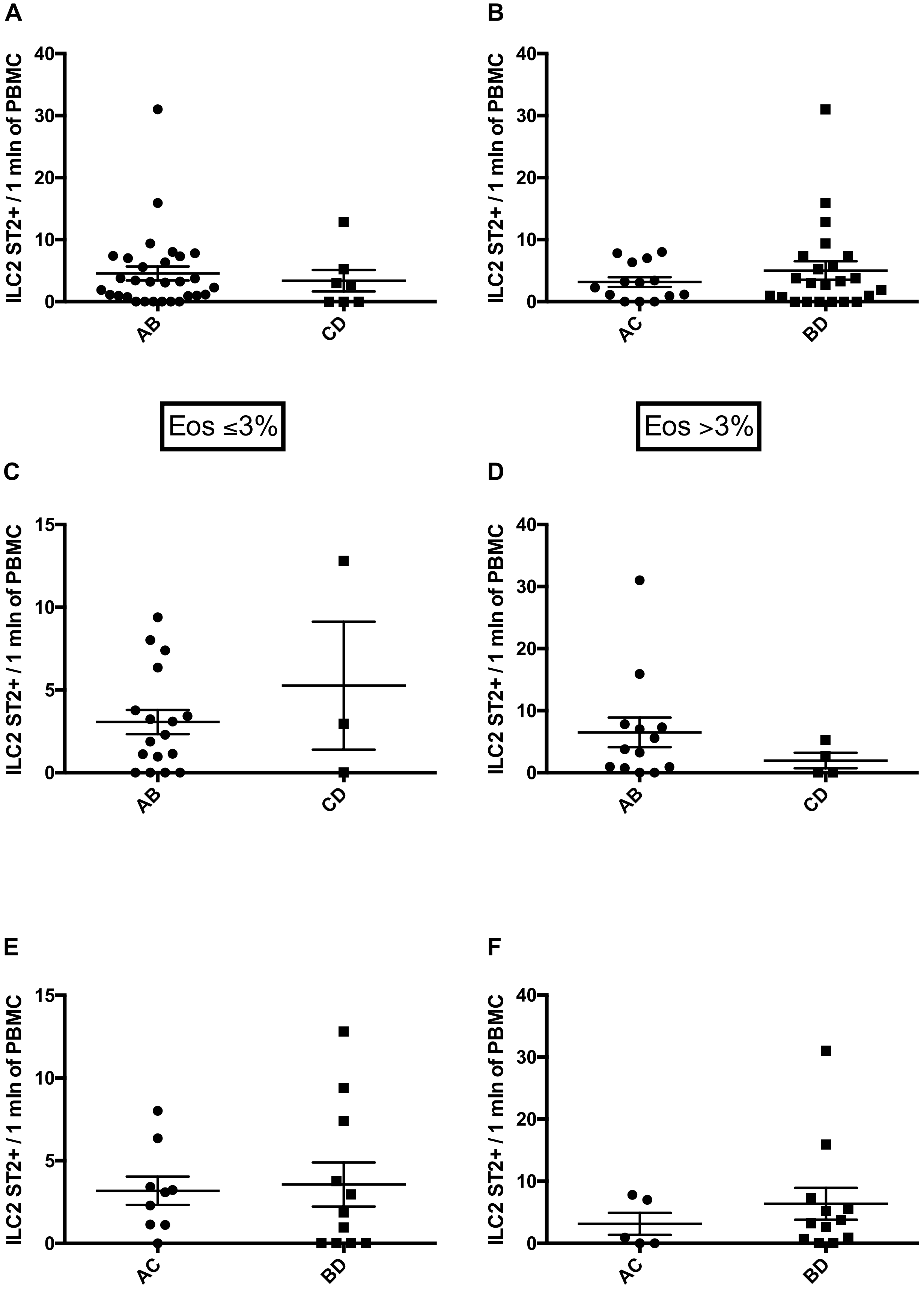
A
B
C
D
E
F
ILC2 ST2+ / 1 mln of PBMC
0
5
10
15
20
30
40
AB
CD
AC
BD
Eos ≤3%
Eos >3%
