# Supplementary figures and images for: Expression of Epithelial Alarmin Receptor on Innate Lymphoid Cells Type 2 in Eosinophilic Chronic Obstructive Pulmonary Disease
Source: Adv Respir Med. 2024 Oct 18;92(5):429–43. doi: 10.3390/arm92050039 (PMC11505438; doi:10.3390/arm92050039)

**A**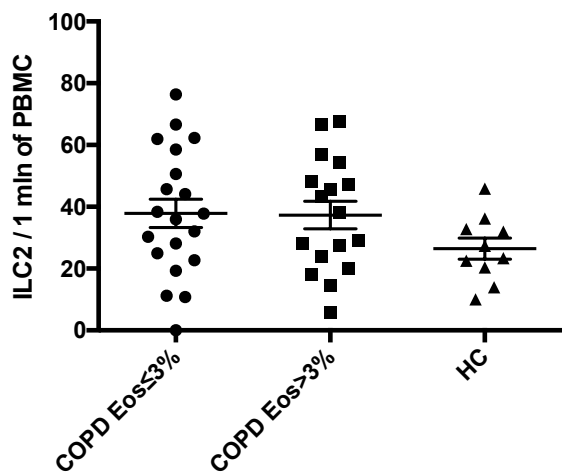**B**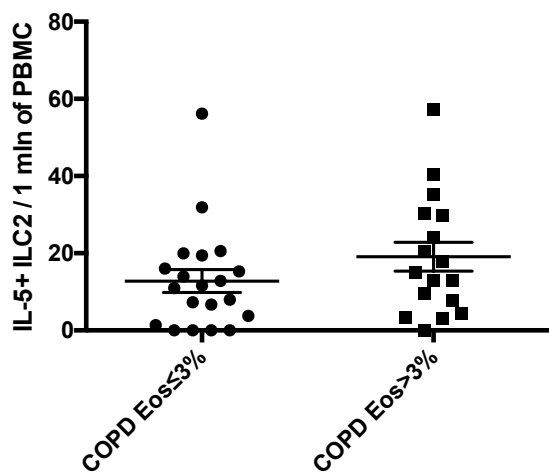**C**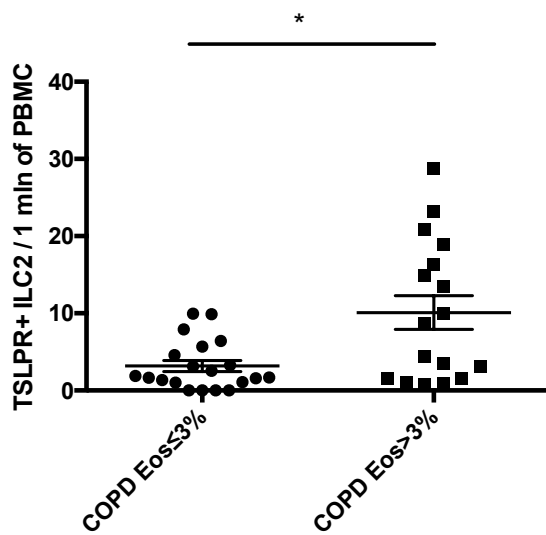**D**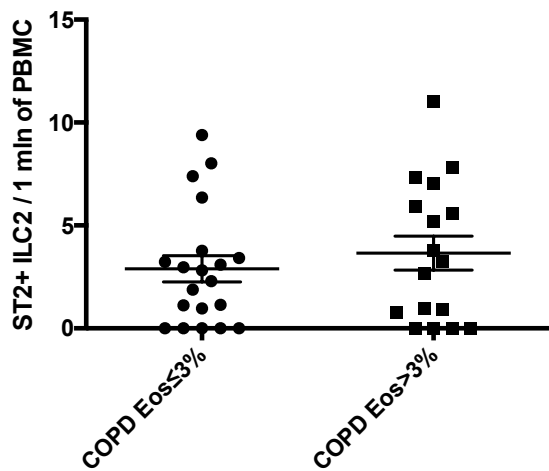**E**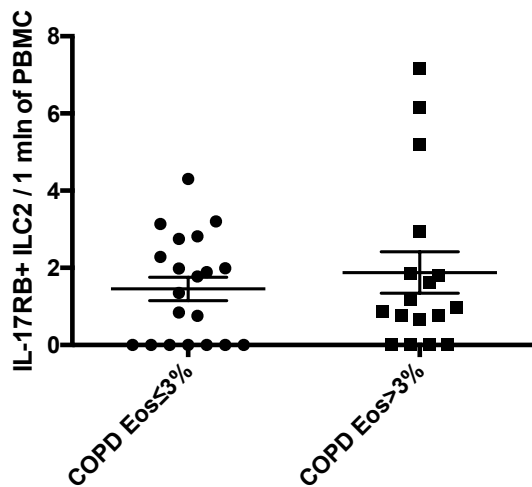

Supplement: Supplementary file 1 [file arm-92-00039-s001.zip › Supplementary Figure 1.pdf]

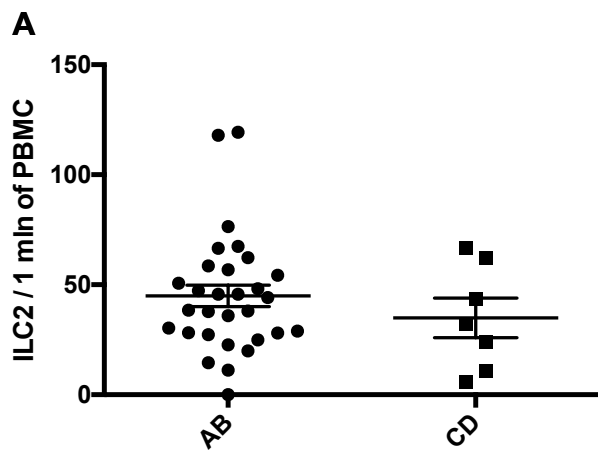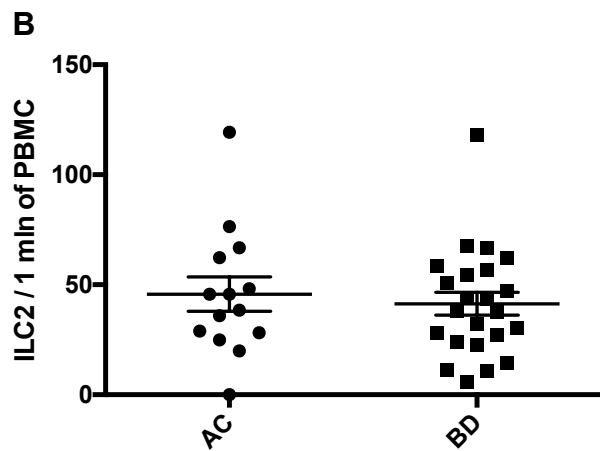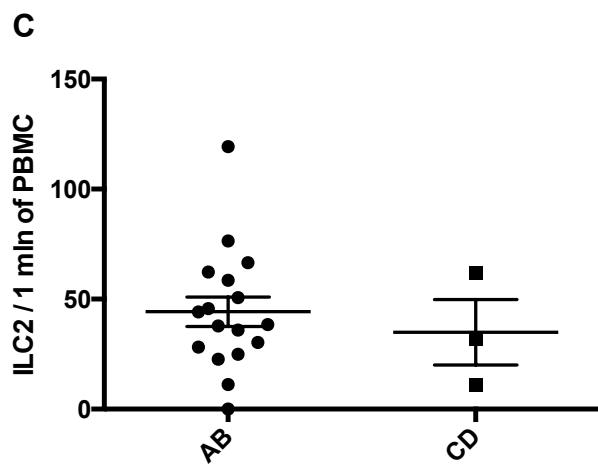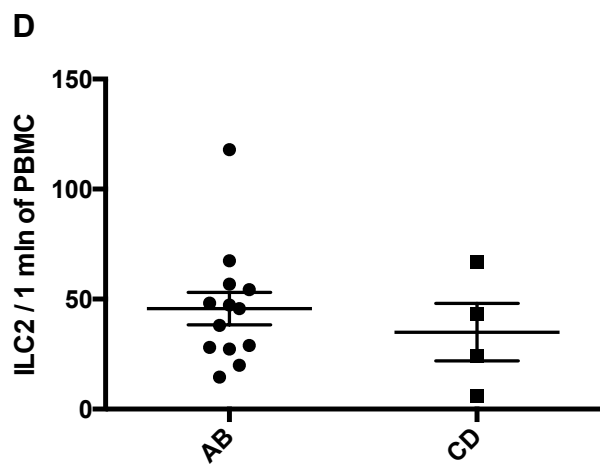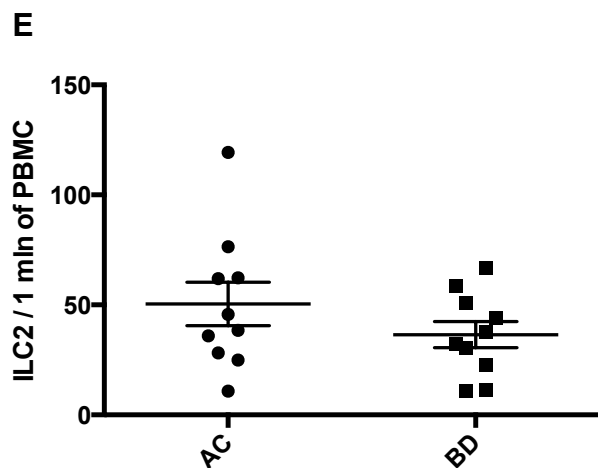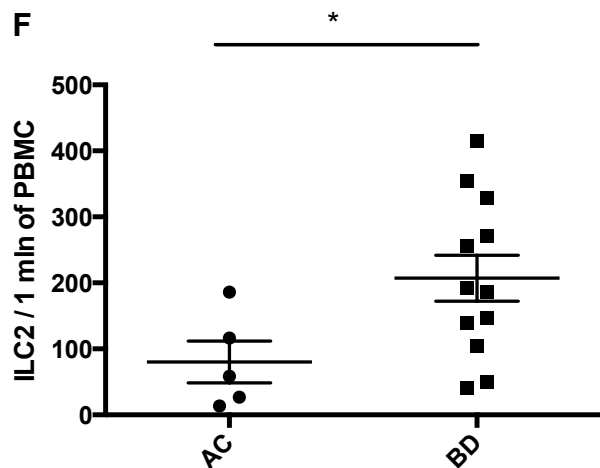

Supplement: Supplementary file 1 [file arm-92-00039-s001.zip › Supplementary Figure 2.pdf]

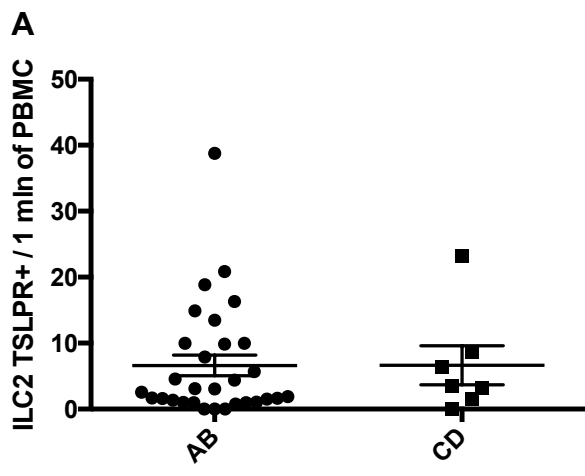

Eos  $\leq 3\%$

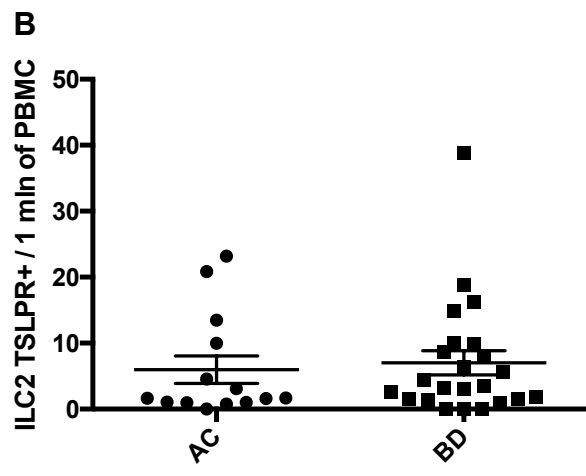

Eos  $> 3\%$

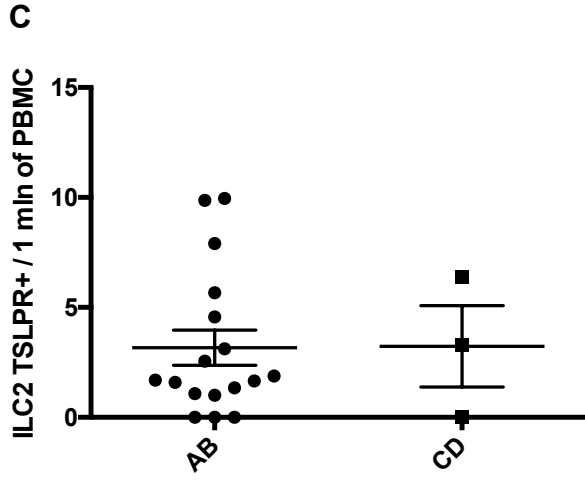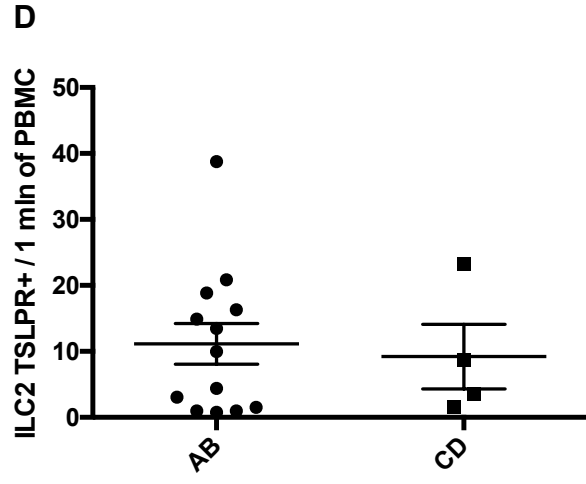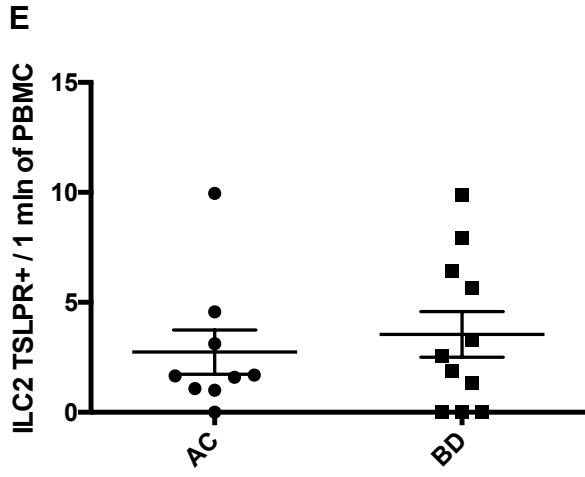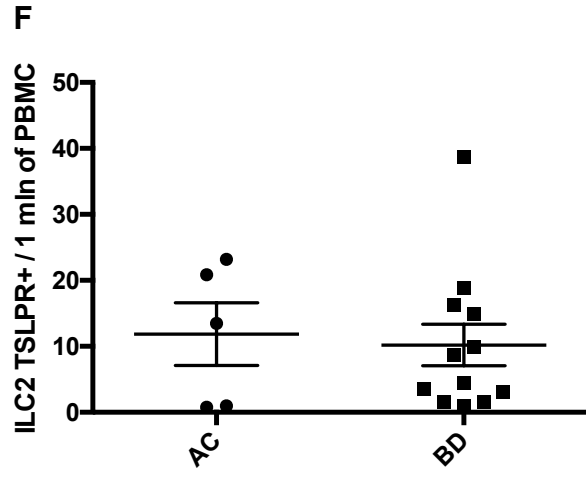

Supplement: Supplementary file 1 [file arm-92-00039-s001.zip › Supplementary Figure 3.pdf]

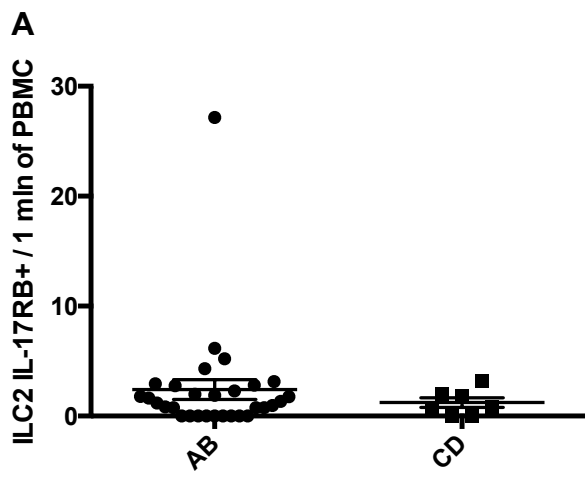

Eos  $\leq 3\%$

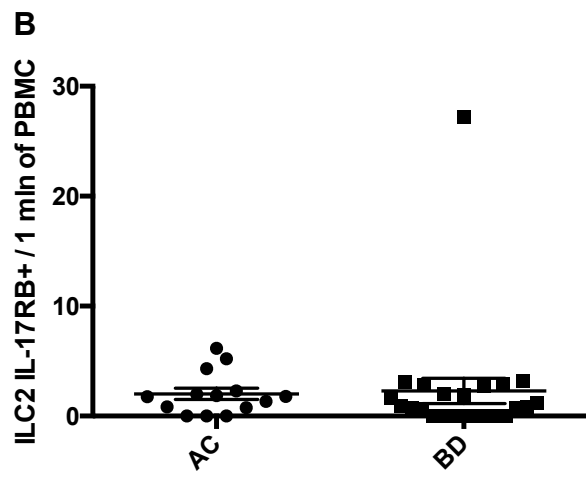

Eos  $> 3\%$

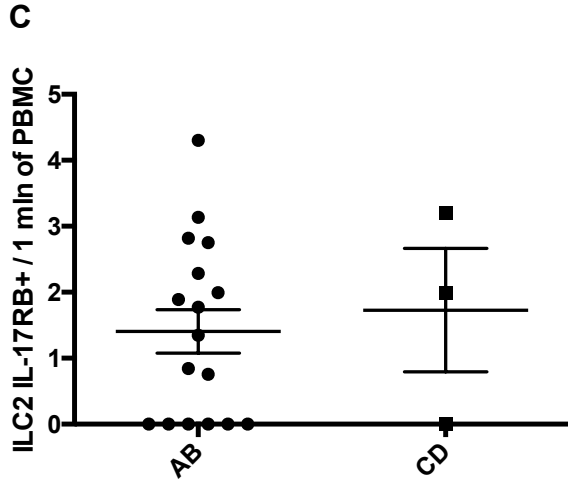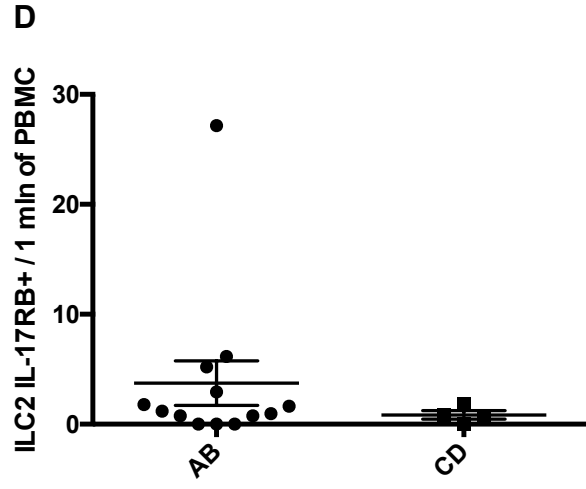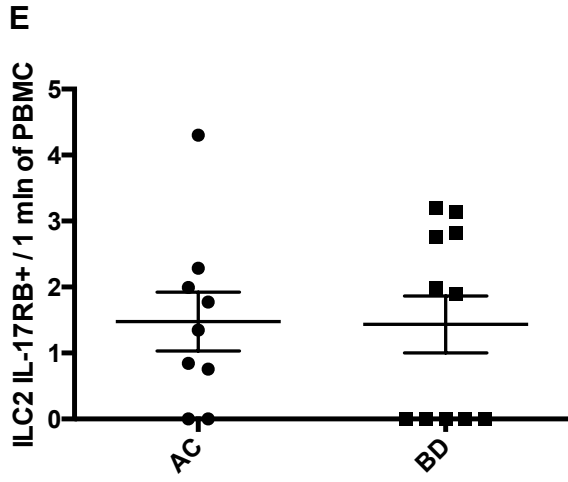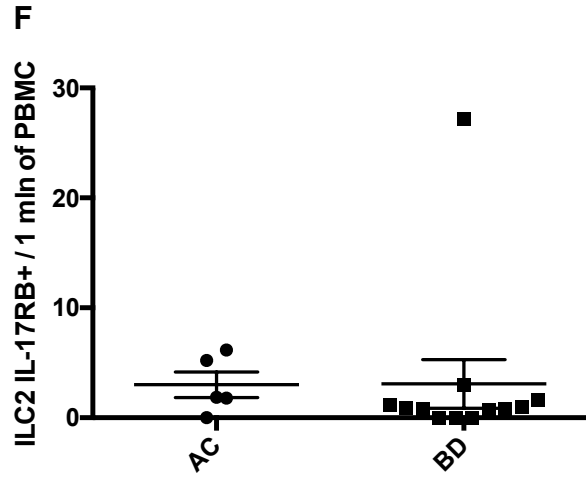

Supplement: Supplementary file 1 [file arm-92-00039-s001.zip › Supplementary Figure 5.pdf]

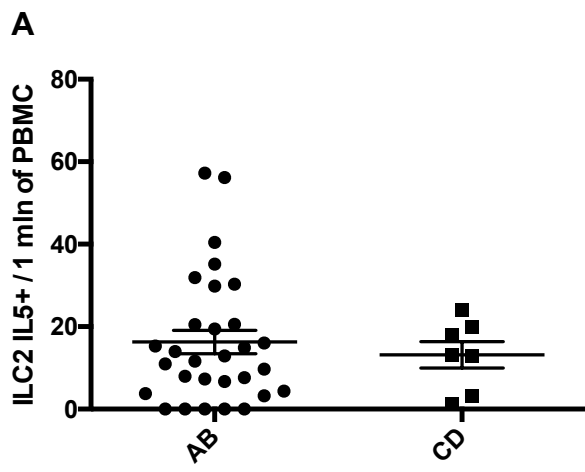

Eos  $\leq 3\%$

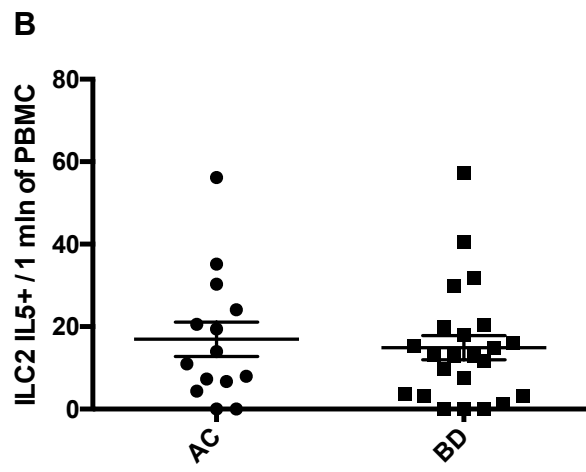

Eos  $> 3\%$

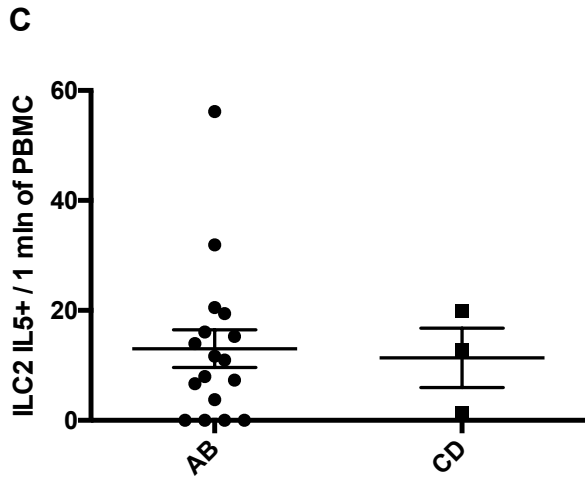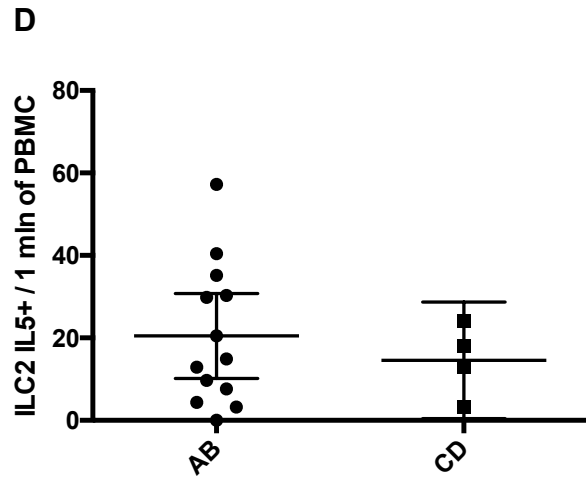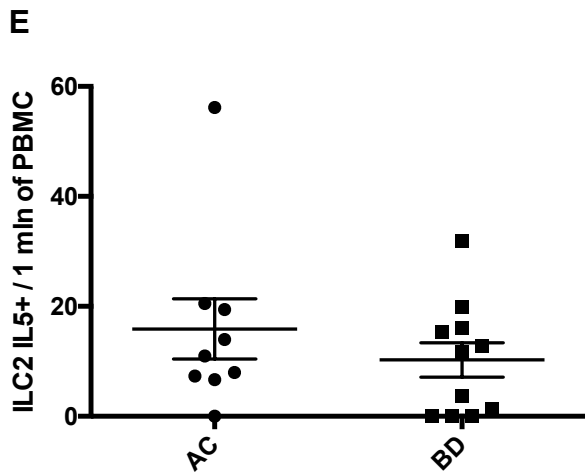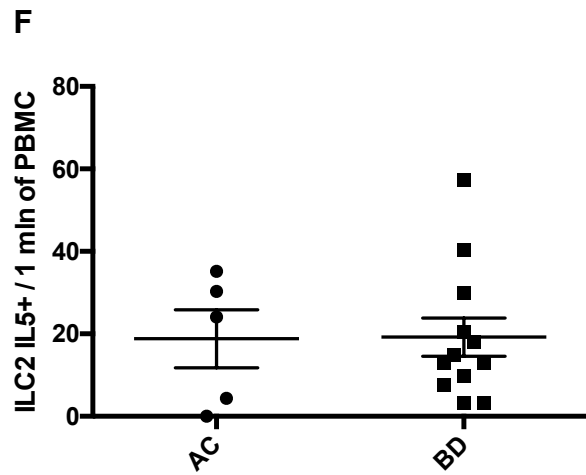

Supplement: Supplementary file 1 [file arm-92-00039-s001.zip › Supplementary Figure 6.pdf]
